# Anaerobic Speed Reserve and Critical Speed as Predictors of 200 m Front Crawl Performance: A Practical Alternative to Physiological Testing in Trained Swimmers

**DOI:** 10.1002/ejsc.70256

**Published:** 2026-10-19

**Authors:** Bryce J. O. Lanigan, Martyn J. Binnie, Peter Peeling, Brendan R. Scott, Jeremiah J. Peiffer, Brad Wall, Eoghan Trihy, Lachlan Mitchell, David B. Pyne, Paul S. R. Goods

**Affiliations:** ^1^ PHysical Activity, Sport and Exercise (PHASE) Research Group School of Allied Health (Exercise Science) Murdoch University Perth Western Australia Australia; ^2^ Western Australian Institute of Sport Mount Claremont Western Australia Australia; ^3^ School of Human Sciences (Exercise and Sport Science) The University of Western Australia Perth Western Australia Australia; ^4^ Centre for Healthy Ageing Health Futures Institute Murdoch University Perth Western Australia Australia; ^5^ Victorian Institute of Sport Albert Park Victoria Australia; ^6^ Research Institute for Sport and Exercise University of Canberra Bruce Australian Capital Territory Australia

**Keywords:** maximal aerobic speed, maximal sprint speed, middle distance, performance modelling

## Abstract

Anaerobic speed reserve (ASR) and critical speed (CS) offer practical, performance‐based estimates of physiological tolerances, and can be derived from maximal aerobic speed (MAS) and maximal sprint speed (MSS) in swimming. We evaluated the predictive capabilities of ASR and CS for 200 m front crawl performance in trained swimmers, and whether the addition of physiological markers could improve model accuracy. Seventeen trained male (*n* = 8) and female (*n* = 9) swimmers completed three maximal front crawl time‐trials (50, 200, and 400 m) on different days, with 50 m (MSS) and 400 m (MAS) used to determine ASR and CS. Blood gas variables, blood lactate concentration (BLa), peak oxygen consumption (VO_2peak_), blood bicarbonate concentration (HCO_3_), and blood pH were measured pre‐ and post‐trial. Linear regression models were developed, with multivariate analysis of variance assessing the impact of distance on all variables. MSS was the best predictor of 200 m performance (*R* ^2^ = 0.83), followed by CS (*R* ^2^ = 0.69), whereas ASR provided moderate predictive capabilities (*R* ^2^ = 0.36). The addition of CS to MSS provided our strongest two‐factor model (*R* ^2^ = 0.93), decreasing mean absolute error (MAE) from 2.41 to 1.62 s, closely followed by MSS + ASR (*R* ^2^ = 0.92, MAE = 1.74 s). Physiological markers of aerobic (VO_2peak_: *r* = 0.44, *p* > 0.05) and anaerobic capacity (ΔHCO_3_: *r* = 0.54; ΔpH: *r* = 0.48; and ΔBLa: *r* = 0.57, all *p* < 0.05) were only moderately correlated with 200 m front crawl performance, and didn't meaningfully improve regression models. MSS provided the highest prediction of 200 m times, whereas ASR and CS provide practical alternatives to the collection of physiological variables which further distinguish performance potential in swimming cohorts.

## Highlights

- Maximal sprint speed was the highest individual predictor of 200 m front crawl performance, illustrating its importance in middle‐distance swimming events, with the addition of CS further strengthening our model.
- Aerobic and anaerobic physiological markers did not meaningfully improve predictive models, suggesting that performance modelling (which offers greater accessibility for practitioners) is a practical and suitable alternative for identifying middle‐distance swimming potential.
- The determination of ASR, CS, MSS, and MAS can be all be performed simultaneously via two commonly performed time trial distances (50 and 400 m) allowing for a time‐efficient estimation of metabolic capacities and performance potential in mixed energy system events.

## 1 Introduction

Aerobic and anaerobic metabolic pathways both play substantial roles in meeting the high energy demands of middle‐distance freestyle swimming (Almeida et al. 2020; Figueiredo et al. 2011). The 200 m freestyle (with front crawl the typical stroke performed) is one such event, with approximately 35% anaerobic and 65% aerobic contributions, with well‐developed capacities in both systems essential for performance (Capelli et al. 1998; Figueiredo et al. 2011; Troup 1991). Performance in the 200 m front crawl is then further mediated by complex interactions between metabolic and mechanical factors (Zamparo et al. 2020), meaning that individuals with different phenotypes can be successful in similar events (Sandford and Stellingwerff 2019). Understanding the interplay between these factors helps tailor training interventions to individual profiles and is therefore of interest to practitioners to ensure training methods employed have the greatest impact (Sandford et al. 2021). To determine performance potential, a combination of both performance and physiological measures is easily accessible in many sports; however, the ability to collect regular and accurate physiological data in swimming cohorts is made difficult by the aquatic nature of the sport (Fernandes et al. 2013). There is a critical need to assess the validity of more accessible methods for quantifying middle‐distance performance potential in swimmers.

Anaerobic speed reserve (ASR; the difference between maximal sprint speed [MSS] and maximal aerobic speed [MAS]) and critical speed (CS; the highest sustainable speed which can be maintained in a physiological steady‐state) are performance‐derived models that offer a practical method for estimating the metabolic impact of different exercise intensities (Dalamitros et al. 2015; Sanders and Heijboer 2019; Sandford et al. 2021). The ASR model is calculated from two distinct landmarks (MAS and MSS) and therefore only requires the completion of two maximal time trials (Dalamitros et al. 2015; Sandford et al. 2019). Estimations of CS are mathematical model‐dependent (Zacca et al. 2010), but utilising the slope of the regression line between 50 and 400 m swim trials offers practical appeal and has been demonstrated as both a valid and reliable method (Dalamitros et al. 2015; Wakayoshi et al. 1992). Swimming coaches and scientists may prefer this approach, especially when working with large squads, as it allows both ASR and CS to be calculated from the same simple performance measures, utilising two familiar race distances (50 and 400 m).

While there is limited research utilising the ASR in swimming, this construct has been explored in sporting events with similar metabolic demands (e.g., 800 m track running; (Sandford et al. 2019)). An individual athlete's ASR helps to explain variance in tolerance to high‐speed exercise (Buchheit et al. 2012; Sandford et al. 2021) and is an important component of performance in 800 m running events (Sandford et al. 2019). However, in the latter investigation, MSS provided the strongest predictive capability (Sandford et al. 2019); while other studies have failed to observe a strong relationship between middle‐distance running (800 and 1500 m) and ASR alone (del Arco et al. 2023). Collectively, these findings provide theoretical utility for the use of ASR in middle‐distance swimming, given the similarity in metabolic demands to 800 m running, but also suggest there may be value in exploring other performance determinants alongside ASR for mixed energy system events.

MSS is a critical factor in swimming, especially given the increased relative importance of energy cost to overall performance (Zamparo et al. 2020), and can be calculated from the same 50 m sprint trial used to determine CS and ASR in combination with a 400 m trial (Dalamitros et al. 2015). Given that swimming performance is mediated by the ratio of maximal metabolic power (sum of anaerobic and aerobic energy production) to energy cost (the mechanical efficiency with which the energy is expressed) (Zamparo et al. 2020), assessment of the ASR may also provide strong utility for predicting middle‐distance swimming performance potential, as it expresses both aerobic (MAS) and anaerobic (ASR) capacities relative to mechanical output (speed/velocity). Assessment of CS has also provided utility in talent identification for elite junior swimmers, especially for 200 m events where aerobic fitness is critical to performance (Mitchell et al. 2018). However, it remains unclear whether performance‐derived models can explain as much of the variance in middle‐distance swimming without the addition of aerobic (e.g., peak oxygen uptake [VO_2peak_]) or anaerobic (e.g., blood lactate [BLa], pH, bicarbonate [HCO_3_]) physiological markers, which are typically collected as part of the testing batteries for land‐based, mixed energy system athletes.

The aim of this study was to determine whether ASR and CS models could predict 200 m front crawl swimming performance times. Further, we sought to investigate whether the addition of selected physiological measures could increase the predictive capabilities of these models. We hypothesised that MSS would be the strongest predictor, and that ASR would also be moderately to highly related to 200 m front crawl performance, whereas CS would provide moderate predictive capabilities. The addition of aerobic (VO_2peak_) and anaerobic physiological measures (HCO_3_, BLa, and pH) to regressions were hypothesised to further increase model performance.

## 2 Materials and Methods

### 2.1 Participants

Seventeen trained (tier 2) (McKay et al. 2022) male (*n* = 8, age: 19.8 ± 3.1 y, mass: 76.2 ± 9.9 kg, height: 180.0 ± 8.1 cm, wingspan: 1.83 ± 0.08 m) and female (*n* = 9, age: 19.2 ± 2.5 y, mass: 68.1 ± 7.2 kg, height: 1.71 ± 0.07 m, wingspan: 1.74 ± 0.07 m) swimmers were recruited from state sporting institute scholarship and pathway programmes. Participants were informed of the nature of the study and associated risks of participation, before providing signed consent, with parent/guardian signed consent obtained where subjects were under the age of 18 years. Ethical approval was provided from the host institution's Human Research Ethics Committee (Reference: 2024/053).

### 2.2 Experimental Design

Participants attended three pool testing sessions, each separated by at least 48 h, and were instructed to avoid alcohol consumption and strenuous exercise for 24 h prior to each visit. All swimming time trial protocols were already familiar to athletes, with additional physiological measures recorded pre‐ or post‐swim, having no impact on performance. Testing was conducted in a standard 50 m Olympic swimming pool, with participants undertaking their typical race warmup (∼1000 m comprising aerobic and variable pace intervals) and replicating this for each trial. A single bout of maximal front crawl swimming of either 50, 200, or 400 m in distance was then conducted in a randomised order from a wall‐push start (to ensure swimming speeds used in the ASR and CS calculations were specific to free swimming). Each trial was conducted at the same time of day, with lane conditions controlled. Stroke rate was measured via video post‐processing throughout exercise, whereas BLa, VO_2peak_, HCO_3_, pH, rating of perceived exertion (RPE), and partial pressure of oxygen and carbon dioxide (pO_2_, pCO_2_) were collected pre‐ and post‐exercise. Both CS and ASR models were then calculated from performance times in the 50 and 400 m events. We specifically looked to assess whether regression models of time ∼ ASR, time ∼ CS, time ∼ MSS, and time ∼ MAS could predict 200 m swimming performance, as a practical profiling technique for swim coaches. Further, it was then explored whether addition of physiological markers could improve these models, or if the combination of these factors could further distinguish performance levels (time ∼ ASR + CS, time ∼ ASR + MSS, time ∼ ASR + MAS, time ∼ CS + MSS, and time ∼ MSS + MAS). Given the conceptual overlap of these performance derived models and their inherent collinearity, we limited our primary regression models to two factors to address potential inflation or variable redundancy, with models exceeding a variation inflation factor (VIF) of 5 excluded from our analysis.

### 2.3 Procedures

Performance times were assessed through video analysis (Kinovea, 2023.1.2) recorded with a waterproof camera (Hero 12 Black, GoPro, California, USA), from last foot contact to first hand touch, with stroke rates averaged over 3 cycles at the 25m mark of the pool every lap. MSS was calculated from the total completion time of the 50 m swim, and MAS from the average speed maintained between 50 and 350 m of the 400 m trial (Dalamitros et al. 2015). Finally, ASR was calculated as the difference between speeds obtained during the 50 m (MSS) and 400 m (MAS) swims, respectively, whereas CS was calculated from the slope of the regression line between each of the swim distances and times (50 and 400 m) (Dalamitros et al. 2015).

A backwards extrapolation technique was employed to assess VO_2peak_ (Costill et al. 1985; Monteiro et al. 2020), through a single 20 s collection of expired ventilation immediately post‐exercise via the Douglas bag method (Hellard et al. 2018; Leger et al. 1980), with volume assessed using a spirometer (Tissot Gasometer, Collins, 120 L, 1967). A face mask, with a two‐way valve attached directly to the Douglas bag, was applied immediately post‐swim (as soon as the head breached the water and before first expiration) with swimmers instructed to utilise their typical race breathing patterns and not to exhale after touching the wall until the mask was secured. To prevent potential leaks, the face mask was firmly applied with assistance from a researcher, with a secondary observer checking for visible deformity from the face. Three 100 mL samples were collected using a syringe (Monoject 140 mL Luer Lock, Covidien) with a three‐way valve to allow for analysis of O_2_ and CO_2_ concentrations via a metabolic measurement system (TrueOne, ParvoMedics, Salt Lake City, United States), which had been calibrated to manufacturer instructions. Each sample had 100 mL flushed through the three‐way valve before collection to ensure dead space in the assessed sample was minimised. This volume was then accounted for in the final calculation.

VO_2peak_ was calculated using an average from all three samples via the following equation:

$${\text{VO}}_{2}=\left(V_{\dot{1}}\cdot F_{I}\cdot O_{2}\right)-\left(V_{\dot{E}}\cdot F_{E}O_{2}\right)$$

and expressed relative to each participant's body mass (mL.min^−1^.kg^−1^). A capillary blood sample (95 μL) was obtained from each participant's fingertip pre‐trial, and 1 min after exercise cessation. Samples were immediately inserted into a CG4+ cartridge for analysis of BLa, HCO_3_, pH, pO_2_, and pCO_2_ using a portable point‐of‐care device (i‐STAT, Abbott, Arbroath, United Kingdom). Cartridges were stored (2°C–8°C, or 18°C–30°C for < 60 d) and calibrated as per manufacturer's instruction prior to use. These devices have been assessed for reliability and deemed suitable for field use (Dascombe et al. 2007). Finally, RPE was also collected pre‐exercise and 1‐min post‐exercise.

### 2.4 Statistical Analysis

A series of multiple linear regression models were employed using R Statistical Software (v 4.3.0) to assess whether ASR and CS models, along with physiological variables, could adequately predict performance in the 200 m maximal front crawl swim. An adjusted *R* ^2^ was reported as the outcome measure, with model form as follows: 200 m time = *β*_0_ + *β*_1_ (Variable 1) + *β*_2_ (Variable 2) + *ε*. Forward selection was used for initial models, with adjusted *R* ^2^ reported consistently for all regression models. Despite inherent and unavoidable collinearity of performance models with performance prediction, these variables were included in regression analysis to assess which metrics provided the most use for practitioners in the field. We expected collinearity may be challenging for our analysis. To address these concerns, we ensured model assumptions of linearity and residual distributions weren't breached, and excluded models with VIF > 5 while testing a priori models. Our primary regression models were also limited to two factors to minimise potential inflation of correlations and variable redundancy in our models, with individual correlations for each separate variable also reported in our results and interpretations softened. Initial analysis was conducted with *n* = 14 participants given missing VO_2peak_ data for three participants to ensure fair comparison between models, with subsequent analysis conducted on *n* = 17 participants once VO_2peak_ was confirmed not to improve model performance. Mean absolute error was then calculated for each model through leave one out cross validation to assess improvement of fit after additional measures were added. Analysis of variance (ANOVA) was performed for physiological variables to assess differences between swimming time‐trial distances, with multivariate ANOVA employed for anaerobic measures to account for collinearity. Post‐hoc analysis was conducted using Bonferroni corrections, with effect sizes calculated, and alpha levels set to 0.05.

## 3 Results

Physiological and performance data from each maximal front crawl swim trial are presented in Table 1, with a correlation matrix presented in Table 2. MSS was the highest predictor of 200 m performance time (adjusted *R* ^2^ = 0.83), followed by CS (*R* ^2^ = 0.69), whereas ASR only provided moderate predictive capabilities (*R* ^2^ = 0.36). The combination of MSS and CS (Intercept: *β* = 251, 95% CI: 231.6 to 269.7; MSS: *β* = −33.1, 95% CI: −42.5 to −23.7; CS: *β* = −42.9, 95% CI: −61.1 to −24.6) explained the most variance of our 2‐factor models (adjusted *R* ^2^ = 0.93, VIF = 1.76), coupled with a decreasing mean absolute error (MAE) from 2.41 to 1.62 s, closely followed by MSS + ASR (Intercept: *β* = 248, 95% CI: 227.86 to 269.06; MSS: *β* = −74.1, 95% CI: −89.28 to −58.99; ASR: *β* = 42.1, 95% CI: 21.44–62.74) (adjusted *R* ^2^ = 0.92, MAE = 1.74 s, VIF = 3.82). The addition of CS, ASR, and ΔpH to MSS in our optimised (but overfitted) model only trivially improved our predictions, accounting for 95% of variance in 200 m front crawl performance and decreasing mean absolute error from 2.41 to 1.46 s. Inspection of residual distribution and homogeneity of variance demonstrated no violation of assumptions.

**TABLE 1:** Physiological and performance responses of *n* = 17 trained swimmers to 50, 200, and 400 m maximal front crawl swims, mean (standard deviation; 95% confidence interval).

Variable	Units	50 m	200 m	400 m	50–200 m	50–400 m	200–400 m
Mean speed	m.s.^−1^	1.81 (0.13; 0.06)	1.52 (0.08; 0.04)	1.42 (0.07; 0.03)	*p* < 0.001 (*d* = 2.78)	*p* < 0.001 (*d* = 3.59)	*p* = 0.013 (*d* = 1.34)
VO_2peak_	mL.min^−1^.kg^−1^	53.12 (9.88; 4.7)	57.79 (9.6; 4.86)	59.11 (9.6; 5.15)	*p* = 0.970 (*d* = −0.36)	*p* = 0.500 (*d* = −0.48)	*p* = 1 (*d* = −0.13)
ΔpH	n/a	0.16 (0.06; 0.03)	0.27 (0.07; 0.04)	0.24 (0.07; 0.03)	*p* < 0.001 (*d* = −1.52)	*p* = 0.006 (*d* = −1.17)	*p* = 0.704 (*d* = 0.40)
ΔHCO_3_	mmol.L^−1^	7.32 (2.6; 1.23)	11.67 (2.44; 1.16)	10.51 (2.79; 1.33)	*p* < 0.001 (*d* = −1.67)	*p* = 0.004 (*d* = −1.14)	*p* = 0.641 (*d* = 0.43)
ΔBLa	mmol.L^−1^	9.25 (3.08; 1.47)	13.88 (3.19; 1.52)	12.04 (3.43; 1.63)	*p* < 0.001 (*d* = −1.43)	*p* = 0.056 (*d* = −0.83)	*p* = 0.342 (*d* = 0.54)
SR	spm	52.3 (4.86; 2.31)	39.99 (3.95; 1.88)	36.07 (4.09; 1.95)	*p* < 0.001 (*d* = 2.70)	*p* < 0.001 (*d* = 3.50)	*p* = 0.041 (*d* = 0.94)
RPE	(0‐20)	14.82 (1.72; 0.82)	17.22 (1.38; 0.66)	16.91 (1.34; 0.64)	*p* < 0.001 (*d* = −1.48)	*p* = 0.001 (*d* = −1.30)	*p* = 1 (*d* = 0.22)
pO_2_	mmHg	107 (14.79; 7.03)	113.41 (8.95; 4.26)	113.23 (6.80; 3.23)	*p* = 0.292 (*d* = −0.51)	*p* = 0.320 (*d* = −0.53)	*p* = 1 (*d* = 0.02)
pCO_2_	mmHg	35.58 (3.25; 1.54)	33.42 (3.80; 1.81)	34.09 (3.25; 1.54)	*p* = 0.249 (*d* = 0.59)	*p* = 0.687 (*d* = 0.44)	*p* = 1 (*d* = −0.18)

*Note:* Significant differences (*p* < 0.05 after Bonferroni adjustments) and effect size (Cohen's d) between distances displayed in *post hoc* tests are indicated for each variable.

Abbreviations: BLa, Blood lactate; HCO_3_, Bicarbonate; pCO_2_, partial pressure of carbon dioxide in the blood; pO_2_, partial pressure of oxygen in the blood; RPE, Rating of perceived exertion; SR, Stroke rate; VO_2peak_, Peak oxygen uptake.

**TABLE 2:** Correlation matrix for performance time, VO_2peak_, MSS, MAS, ASR, CS, ΔpH, ΔHCO_3_, ΔBLa, pO_2_, and pCO_2_ for 200 m front crawl.

Pearson's *r* (*p*)	Time (s)	VO_2peak_	MSS	MAS	ASR	CS	ΔpH	ΔHCO_3_	ΔBLa	pO_2_
VO_2peak_ (mL.min^−1^.kg^−1^)	−0.44 (0.082)									
MSS (m.s.^−1^)	−0.92 (< 0.001)	0.36 (0.176)								
MAS (m.s.^−1^)	−0.86 (< 0.001)	0.40 (0.129)	0.70 (0.002)							
ASR (m.s.^−1^)	−0.63 (0.007)	0.20 (0.456)	0.86 (< 0.001)	0.24 (0.357)						
CS (m.s.^−1^)	−0.84 (< 0.001)	0.42 (0.103)	0.66 (0.004)	0.99 (< 0.001)	0.18 (0.487)					
ΔpH	−0.49 (0.048)	0.09 (0.728)	0.41 (0.106)	0.25 (0.331)	0.37 (0.140)	0.26 (0.307)				
ΔHCO_3_ (mmol.L^−1^)	−0.54 (0.026)	0.19 (0.491)	0.54 (0.026)	0.29 (0.268)	0.53 (0.029)	0.29 (0.265)	0.89 (< 0.001)			
ΔBLa (mmol.L^−1^)	−0.57 (0.016)	0.27 (0.322)	0.58 (0.013)	0.28 (0.279)	0.60 (0.011)	0.28 (0.286)	0.90 (< 0.001)	0.97 (< 0.001)		
pO_2_ (mmHg)	−0.45 (0.07)	0.10 (0.703)	0.43 (0.082)	0.32 (0.218)	0.36 (0.151)	0.30 (0.236)	0.59 (0.012)	0.49 (0.047)	0.49 (0.045)	
pCO_2_ (mmHg)	0.19 (0.458)	0.09 (0.732)	−0.08 (0.772)	−0.03 (0.911)	−0.08 (0.753)	−0.02 (0.938)	−0.36 (0.150)	−0.21 (0.414)	−0.21 (0.411)	−0.24 (0.349)

*Note:* Significant differences (*p* < 0.05 after Bonferroni adjustments) between distances displayed in *post hoc* tests are indicated.

Abbreviations: ASR, Anaerobic speed reserve; BLa, Blood lactate; CS, Critical speed; HCO_3_, Bicarbonate; MAS, Maximal aerobic speed; MSS, Maximal sprint speed; pCO_2_, partial pressure of carbon dioxide in the blood; pO_2_, partial pressure of oxygen in the blood; VO_2peak_, Peak oxygen uptake.

Effect of swimming distances on all variables is reported in Table 1, with post‐hoc analysis revealing despite significant differences in mean speed for each trial, no difference was observed for VO_2peak_ between any distances (*p* ≥ 0.500), with these measures illustrated in Figure 1. Substantial differences were observed between the 50 m and both 200 and 400 m maximal swims for ΔHCO_3_ and ΔpH, (*p* < 0.001, *p* = 0.004; and *p* < 0.001, *p* = 0.006, respectively), but not between 200 and 400 m (*p* = 0.641; and *p* = 0.704, respectively). The ΔBLa was also substantially different between the 50 and 200 m time trial distances (*p* < 0.001).

**FIGURE 1:**
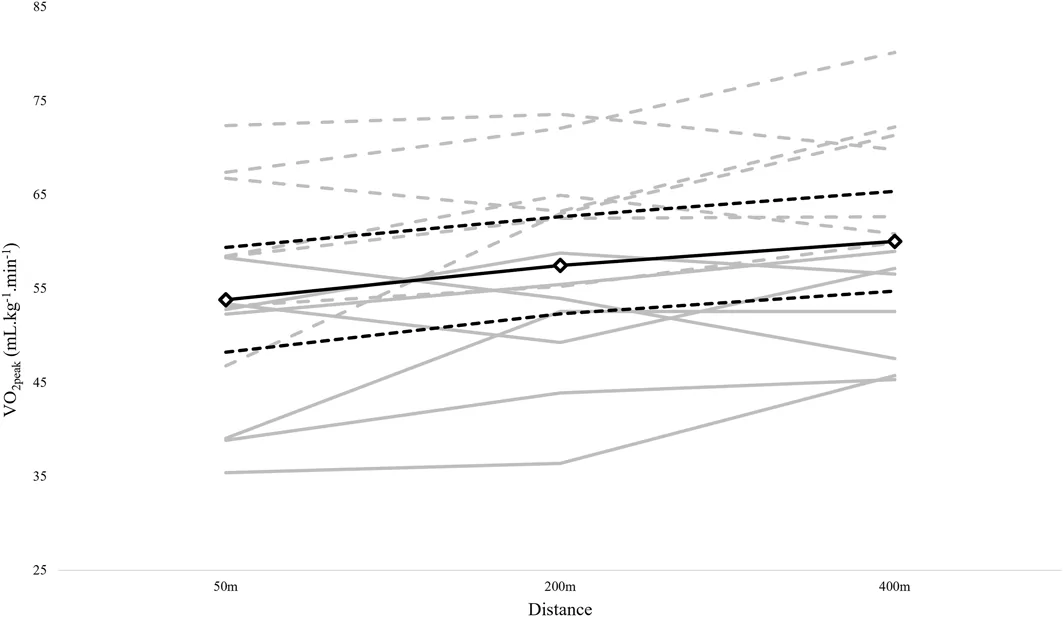
Trained swimmers (*n* = 17) VO_2peak_ response to 50, 200, and 400 m maximal front crawl swims, as measured by back‐extrapolation technique. Light grey lines indicate individual athletes (solid lines: female; dashed lines: males) with black line indicating means and standard deviations.

## 4 Discussion

We evaluated whether performance‐derived physiological models (ASR and CS) could adequately predict 200 m front crawl performance, and whether the inclusion of physiological variables could improve predictive capabilities. Maximal sprint speed derived from the 50 m front crawl was the highest single predictor of 200 m front crawl time, accounting for 83% of the variance in performance. By also assessing 400 m front crawl performance, CS and ASR were calculated and added to MSS in separate two‐factor models, which reduced MAE (from 2.41 s to 1.62 and 1.74 s) and increased predictive capabilities (93% and 92%, respectively) for estimating 200 m front crawl time. These models outperformed others which included physiological variables. Collectively, these outcomes highlight the potential utility of performance‐derived models for interpolating 200 m front crawl time, based on simple assessments of 50 and 400 m swimming performance.

The importance of MSS to middle‐distance front crawl performance is unsurprising, given athletes who are able to produce the highest short sprint velocities are likely be able to translate this to faster performance times (Sandford et al. 2019). Further, the relatively large roles that hydrodynamic drag and propulsive efficiency play in maximal swim velocity (Zamparo et al. 2020) partially explain our finding that the interplay between mechanical efficiency and physiological factors (MSS) had a higher impact on swim performance than metabolic factors alone. Even over longer time trial durations (> 2 min for 200 m in this investigation), technically proficient swimmers can maintain their velocity more efficiently, by applying greater propulsive forces, and maintaining a streamline position, which incurs lower hydrodynamic resistance and metabolic cost (Gatta et al. 2018; Zamparo et al. 2020). This assertion is supported by our observations, with none of the measured physiological variables able to independently predict 200 m front crawl swim times, or outperforming our performance‐derived measures which inherently incorporate these biomechanical factors. However, whether this result could be replicated in a homogenous group of elite swimmers, with greater technical proficiency, requires further investigation in that cohort. Further, because maximal metabolic power for 200 m front crawl relies heavily on energy produced through both aerobic and anaerobic pathways (Figueiredo et al. 2011; Hellard et al. 2018), anaerobically dominant “sprint” athletes are still well suited to events of this duration. Similar observations were made by Sandford et al. (2019) in 800 m running cohorts, with MSS strongly correlated with faster performance times. This however was conducted with an elite cohort and attributed to the surging nature of 800 m running events, with results in our investigation more likely linked with technical proficiency given the self‐paced nature of 200 m swimming. In line with our hypothesis, it is therefore unsurprising that individuals who demonstrated the highest MSS also performed well in the 200 m front crawl.

The relationship between 200 m front crawl performance time and both ASR (adjusted *R* ^2^ = 0.36) and CS (adjusted *R* ^2^ = 0.69) was moderate, yet the potential to further distinguish performance times warranted inclusion in our models. When interpreting these results, it is important to consider the inherent conceptual overlap between these predictors' which may inflate apparent model performance, as demonstrated by the collinearity of performance models. However, we restricted our primary regression models to two factors, to limit redundancy while allowing comparison in potential utility of each method for assessing performance potential in swimming cohorts. While MSS was the highest predictor, given the large energy requirements from both anaerobic and aerobic systems for 200 m front crawl (Figueiredo et al. 2011), it is also unsurprising that performance was mediated by ASR and CS, as they express energy production capabilities from both pathways within the performance metrics. di Prampero's (1986) originally proposed equation (*v*_max_ = E/C) illustrated the importance of the interaction between energy cost (C) and maximal metabolic power (E) for determining maximal swimming velocity (*v*_max_), with performance dictated by the ratio of energy produced to the economy of mechanical expression. Given ASR and CS offer translation of metabolic capacities as a proponent of speed, they therefore provide a higher prediction of an individual's performance and training capabilities, than physiological markers alone. While this interpretation was in line with our second hypothesis, we did not expect to find that none of the aerobic or anaerobic physiological measures collected were able to meaningfully improve our regression model. One possible explanation for limited value of physiological measures is that relationships between ASR, CS, and performance may be more pronounced in more homogenous elite swimmer cohorts, where C is less varied. Given that C is dependent on the interaction between propelling efficiency, mechanical efficiency, and hydrodynamic resistance (drag), swimmers with low drag and high propelling efficiency will perform better than their peers (who may possess similar physiological capacities but higher drag and low propelling efficiency) (Figueiredo et al. 2011). It is likely that in more elite cohorts, who have greater technical proficiency, we might observe more pronounced influences of the relative energetic capacities (whether derived from performance times, such as ASR, MAS, and CS, or from the additional physiological measures we collected here), than MSS in this study. While the swimmers in this investigation were all trained, future studies in this area should aim to recruit elite swimmers from national and international levels to determine whether our findings are generalisable to this cohort.

Despite several significant and predictable differences in anaerobic markers between swim distances, ΔBLa, ΔHCO_3_, and ΔpH provided minimal predictive capabilities of 200 m performance time. Our observation that ASR was moderately correlated with both ΔBLa and ΔHCO_3_ was expected, given ASR provides an estimate of anaerobic capacity, and further demonstrates its utility as a practical assessment tool in swimming cohorts. The inability of VO_2peak_ to increase predictive capabilities can be partially attributed to an unexpected lack of difference in VO_2peak_ between distances, but also justifies the use of performance‐based modelling as a primary assessment tool when monitoring swimming training and performance. This is an encouraging finding given the difficulties associated with collecting physiological data (in particular VO_2peak_) in an aquatic environment. Although other studies have shown that assessment of VO_2peak_ via backwards extrapolation can provide both valid and reliable data (Costill et al. 1985; Zamparo et al. 2000), variations in measures introduced by this technique (e.g., different breathing patterns, potential leaks) may obscure differences between swimmers and limit predictive capabilities. Although mean VO_2peak_ increased across the time trial distances (Figure 1), individual variability was high, with some participants recording higher values in their 50 m trials. This shortcoming was highlighted by the similar values of VO_2peak_ observed between distances (50, 200, and 400 m) despite a logical increase in global oxygen consumption over the longer trial durations included in this investigation. While physiologically justifiable, the inability to distinguish VO_2peak_ between distances decreases confidence in VO_2_ measures via backwards extrapolation, and limits this approach from adding any predictive capabilities.

While in this investigation we demonstrated the utility of performance‐derived models for predicting mixed energy system, middle‐ distance front crawl performance, there are other applications for this work. Quantifying the anaerobic contribution to supramaximal performance and linking it to mechanical output, as illustrated by Sandford et al. (2021), may permit using the ASR to distinguish individual responses to similar training stimuli, particularly where metabolic limitations significantly influence performance. Time to fatigue during high intensity interval training can display a strong relationship with ASR, and further, the personalised prescription of intervals based on ASR can normalise time to fatigue and physiological markers within training sets (Bok et al. 2023; Julio et al. 2020; Thron et al. 2025). Individualising training sessions based on athlete profiles is potentially important to maximise adaptive responses, due to differences in fatigue mechanisms and development rates; ASR provides a practical option for coaches to explore such session individualisation (Sandford et al. 2021). The predictive capabilities of performance‐derived models demonstrated in our study highlight their potential use in prescribing high intensity interval training for swimmers, particularly in the absence widely accepted methods of measuring anaerobic contributions in these exercise domains (Haugen et al. 2018; Sandford et al. 2021). However, this specific aspect was not tested in the current study, and the utility of ASR to individualise training prescription in swimmers warrants further investigation.

Like all studies, this investigation was not without its limitations. While we ensured all participants met the requirements to be considered trained (McKay et al. 2022), a larger sample size inclusive of elite national and international‐level athletes, and swimmers with different specialisations would be useful to broaden the generalisability of these findings. As such, while our observations indicate that ASR and CS modelling can be sufficient for prediction of 200 m performance, physiological measures may still be important for longitudinal monitoring and understanding mechanisms of change. Calculation of CS from short‐distance trials may also function primarily as a performance‐derived index and may not fully represent a sustainable physiological boundary or intensity‐domain transition, given the impact of shorter, largely anaerobic efforts, in estimations. The addition of longer distances to these calculations or sensitivity analysis with alternate distance combinations should be considered for future studies to strengthen interpretations. Future studies should also investigate whether standardising breathing patterns within swimming time trials when utilising the backwards extrapolation technique might reduce variability, which could impact VO_2peak_ determination.

### 4.1 Practical Recommendations

The ASR and CS models provide an accessible method of assessment for estimation of metabolic capacities, with greater predictive capabilities for middle‐distance performance than currently available physiological measures. Given the importance of individualising training programmes to the athlete's physiological profile, ASR and CS modelling provide a practical tool for assessing swimmer's capacities in an aquatic environment (Sandford et al. 2021). Further, MSS should remain a key focus of training for middle‐distance swimmers given its importance to 200 m front crawl performance observed here. While the determination of VO_2peak_ via backwards extrapolation remains a valid and reliable method, our findings suggest that it may not be the best use of practitioner time, as it did not improve the predictive capability of performance‐derived models.

## 5 Conclusion

Our data showed that MSS provided the strongest single predictor of 200 m front crawl performance, while the addition of either CS or ASR substantially improved the models. Physiological markers of aerobic and anaerobic metabolism did not meaningfully improve the predictive model. The determination of ASR and CS via a 50 and 400 m time trial provides a practical approach for identifying performance potential in this mixed energy system event for trained swimmers, noting that it may be especially relevant in swimming cohorts where the collection of traditional laboratory‐based physiological variables is difficult.

## Funding

The authors have nothing to report.

## Ethics Statement

The study was approved by the host institution's Human Research Ethics Committee in accordance with the Declaration of Helsinki (Reference: 2024/053).

## Consent

Participants were provided with information detailing the purpose and requirements of the investigation and gave written informed consent.

## Conflicts of Interest

The authors declare no conflicts of interest.

## Data Availability

The data that support the findings of this study are available from the corresponding author upon reasonable request.
